# Deorphanization and characterization of the ectopically expressed olfactory receptor OR51B5 in myelogenous leukemia cells

**DOI:** 10.1038/cddiscovery.2016.10

**Published:** 2016-05-09

**Authors:** S Manteniotis, S Wojcik, J R Göthert, J Dürig, U Dührsen, G Gisselmann, H Hatt

**Affiliations:** 1 Department of Cell Physiology, Ruhr-University Bochum, Bochum, Germany; 2 Department of Hematology, University Hospital Essen, Essen, Germany

## Abstract

The ectopic expression of olfactory receptors (ORs) in the human body has been of major interest in the past decade. Several studies have reported the expression of ORs not only in healthy tissues such as heart, sperm or skin cells, but also in cancerous tissues of the liver, prostate or intestine. In the present study, we detected the expression of OR51B5 in the chronic myelogenous leukemia (CML) cell line K562 and in white blood cell samples of clinically diagnosed acute myelogenous leukemia (AML) patients by reverse transcription-PCR and immunocytochemical staining. The known OR51B5 ligand isononyl alcohol increased the levels of intracellular Ca^2+^ in both AML patient blood cells and K562 cells. With calcium imaging experiments, we characterized in greater detail the OR51B5-mediated signaling pathway. Here, we observed an involvement of adenylate cyclase and the downstream L-type and T-type calcium channels. In addition, the activation of OR51B5 leads to an inhibition of cell proliferation in K562 cells. In western blot experiments, we found that incubation with isononyl alcohol led to a reduction in p38-MAPK (mitogen-activated protein kinase) phosphorylation that might be responsible for the decreased cell proliferation. In the present study, we characterized the OR51B5-mediated signaling pathway downstream of the activation with isononyl alcohol, which leads to reduced proliferation and therefore provide a novel pharmacological target for CML and AML, the latter of which remains difficult to treat.

## Introduction

Olfactory receptor (OR) genes are known to be expressed mainly in the olfactory epithelium, providing rats and humans with the ability to detect volatile odors in their environments.^1^ In humans, ~1000 different OR genes have been identified, whereas ~400 of these receptors are known to be functional. The chemical ligands for only 10% of the functionally expressed ORs are currently described. New expression analysis showed that the expression of OR genes is not necessarily restricted to the nasal epithelium but can be found in almost all parts of the human body. Unfortunately, the physiological function of ectopically expressed ORs has been shown for only a limited number of receptors.

OR1D2 was the first detected OR to be ectopically expressed in spermatogonia and shown to be involved in chemotaxis.^2^ A few years later, it was demonstrated that an OR-specific odor stimulation led to serotonin release from enterochromaffine cells of the gut via OR activation.^3^ The prostate-specific G-protein-coupled receptor, also known as OR51E2, is highly expressed in prostate cells and in the prostate cancer cell line LNCaP.^4,5^ In 2009, the physiological role of OR51E2 was characterized using the agonist *β*-Ionone in LNCaP cells.^6^ The activation of OR51E2 elicited a rapid Ca^2+^ increase that caused an inhibition of proliferation via mitogen-activated protein kinase (MAPK) phosphorylation. Recently, a study showed that in hepatocellular carcinoma and in the cell line Huh7, the OR1A2 increased intracellular Ca^2+^ levels and reduced cell proliferation after (−)-citronellal treatment.^7^ Additional elucidated roles for ORs in various tissue types include involvement in cell cytokinesis, blood pressure regulation and enzyme secretion in the kidneys.^8,9^ In 2014, it could be shown that OR2AT4 could be detected in the skin and in primary keratinocytes, respectively and activated by Sandalore, a synthetic derivate of Sandalwood.^10^ The activation of OR2AT4 led to increase in proliferation and cell migration and to a faster wound healing. A recent study showed that in AML blood cell samples and in the CML cell line K562 the OR2AT4 led to a decreased proliferation and induced apoptosis after activation with Sandalore.^11^ In addition, erythrocyte stimulation could be observed in Sandalore-incubated K562 cells. According to previous studies intracellular Ca^2+^ can activate several physiological responses, such as differentiation, inhibition of proliferation, apoptosis or chemotaxis, through the activation of downstream mechanisms.^12–14^

In the present work, we used K562 cells as a model for chronic myelogenous leukemia (CML). The K562 cell line was derived from a 53-year-old CML patient in blast crisis.^15^ This commonly used cell line serves as a model to explore the molecular mechanisms of late-stage human myeloid leukemia. The K562 cell line contains primarily myeloid precursor cells and undifferentiated granulocytes and a few amount of erythrocytes.^16,17^ Unfortunately, most CML-infected patients in blast crisis are physically weak. To investigate the mechanisms in native blood cells of myelogenous leukemia patients, we used blood from acute myelogenous leukemia (AML) patients, due to the high levels of undifferentiated blast cells in the peripheral blood of AML patients.

Here, we show that OR51B5 is activated by its known ligand isononyl alcohol in K562 cells and in white blood cells of clinically newly diagnosed AML patients. We validated the expression of OR51B5 in K562 cells and in AML blood samples. The activation of OR51B5 in K562 cells leads to an inhibition of cancer cell proliferation. Our work provides a novel pharmacological target for CML and AML.

## Results

### OR51B5 is expressed in myelogenous leukemia cells

**Figure 1:**
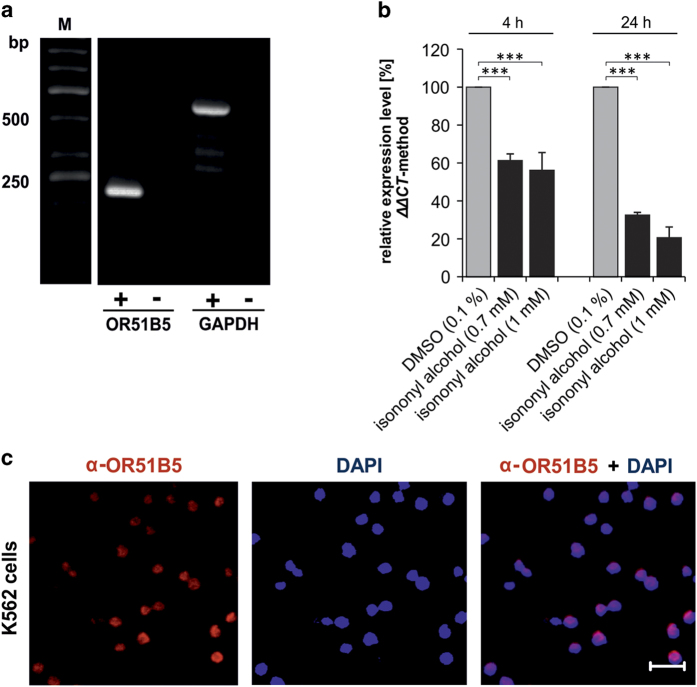
OR51B5 is expressed in the CML cell line K562. (**a**) RT-PCR experiments show the expression of OR51B5 in K562 cells at the expected size of ~230 bp. GAPDH was used as a control. (**b**) qPCR experiments showed that after 4 h isononyl alcohol incubation OR51B5 expression was significantly decreased. The effect was enhanced by longer stimulation time (24 h). (**c**) Immunocytochemical staining with the OR51B5 antibody validates the expression of OR51B5 in K562 cells. DAPI was used to stain the cell nuclei.

In a recent study, using next-generation sequencing we found that OR51B5 is the most highly expressed OR in the chronic myelogenous leukemia cell line K562.^11^ Here, we validated the expression of OR51B5 by reverse transcription (RT)-PCR and immunocytochemical stainings (Figure 1a).

Recently, it was shown using HEK293 cell transfection experiments, that isononyl alcohol is a ligand for OR51B5, which had previously been considered an orphan receptor (personal communication).

To investigate whether the treatment with varying isononyl alcohol concentrations alters the OR51B5 expression level, we incubated K562 cells for a period of time with the odorant and subsequently analyzed the expression level of OR51B5 using qPCR and the ΔΔCT method (Figure 1b). During the incubation of K562 cells with the OR51B5 agonist, the expression of the receptor was downregulated in a time-dependent manner. After 4 h of incubation, 0.7–1 mM isononyl alcohol significantly downregulated the expression of the receptor compared with the control. This effect was enhanced by an increased incubation time with the OR51B5 agonist (up to 24 h). Next, we validated the RT-PCR experiments using immunocytochemical staining (Figure 1c).

### Isononyl alcohol increases the intracellular Ca^2+^ concentration in K562 cells

**Figure 2:**
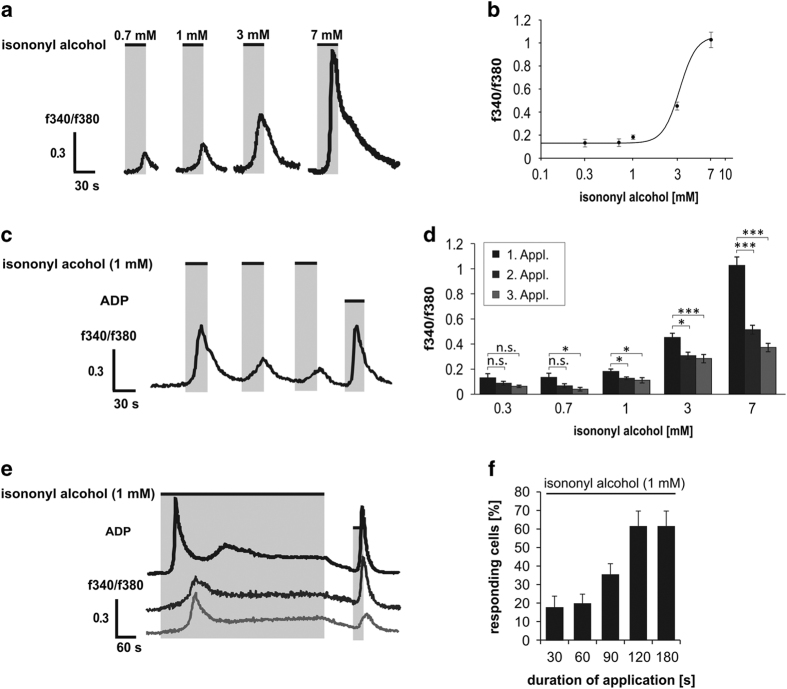
Calcium imaging experiments with the CML cell line K562. (**a**) Application of different concentrations of isononyl alcohol increased the levels of intracellular Ca^2+^. (**b**) The EC_50_ of isononyl alcohol in K562 cells was 3.4±0.2 mM (*n*=16). (**c**) Receptor desensitization was observed after repetitive isononyl alcohol stimulation. (**d**) Summarized results for the receptor desensitization after the application of different isononyl alcohol concentrations. (**e**) Long-term stimulation (6 min) with isononyl alcohol increased the cytosolic Ca^2+^ level within the first 2 min of the application time. ADP was used as a positive control to measure cell viability. (**f**) After 2 min of odor application, a maximum of ~58% of the cells showed an increase in their intracellular calcium levels.

It is well known that the binding of ligands to their specific OR activates an intracellular G-protein-mediated signaling cascade, which leads to an increase in intracellular Ca^2+^.^2,3,6,7^ Here, we sought to investigate if the activation of OR51B5 can similarly increase the intracellular Ca^2+^ level in K562 cells. In Figures 2a and b, we have shown that different concentrations of isononyl alcohol (0.3–7 mM) are able to increase intracellular Ca^2+^ levels to varying degrees. The calculated EC_50_ of isononyl alcohol in K562 cells was 3.4±0.2 mM. After the application of 1 mM isononyl alcohol on K562 cells, we observed a strong receptor desensitization, as has been observed for other ectopically expressed ORs (Figure 2c).^7^ We summarized the mean values for the isononyl alcohol-induced receptor desensitization in Figure 2d. Next, we treated cells with 1 mM isononyl alcohol for 6 min and observed a short increase in intracellular Ca^2+^ during the first 2 min of the odor application (Figure 2e). After the first 2 min, 60% of the cells continued to respond to isononyl alcohol with an increase in their cytosolic Ca^2+^ levels (Figure 2f). After applying repetitively 1 mM isononyl alcohol on K562 we observed a strong receptor desensitization, same like it was observed for other ectopically expressed ORs.

### Isononyl alcohol activates an AC-mediated signaling pathway in K562

**Figure 3:**
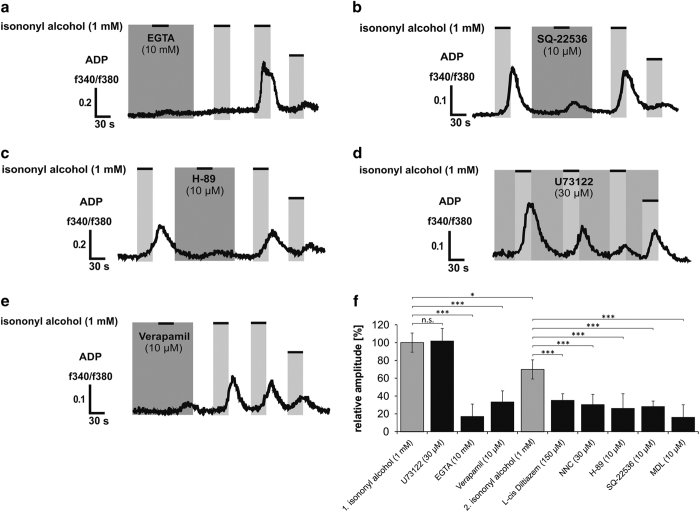
Characterization of the OR51B5-mediated intracellular signaling components. (**a**) Under Ca^2+^-free conditions, an increase in cytosolic Ca^2+^ levels during isononyl alcohol stimulation was almost completely abolished. (**b**) During the co-treatment with isononyl alcohol and the AC inhibitor SQ-22536, no Ca^2+^ signals could be detected. (**c**) The PKA inhibitor H-89 blocked the isononyl alcohol-induced increase in intracellular Ca^2+^. (**d**) The PLC inhibitor U73122 could not significantly block the isononyl alcohol-mediated Ca^2+^ increase. (**e**) L-type calcium channel inhibitor Verapamil abolished the isononyl alcohol-induced Ca^2+^ increase in K562 cells. (**f**) The inhibitor experiments were analyzed relative to the first and second applications of isononyl alcohol.

To further investigate the OR51B5-mediated signaling pathway downstream of isononyl alcohol activation, an extracellular Ca^2+^ chelator was used to determine the source of incoming Ca^2+^ (Figure 3a). In Ca^2+^-free extracellular medium, isononyl alcohol was not able to elicit any Ca^2+^ response in K562 cells. After rewashing the cells with a Ca^2+^ containing Ringer’s solution, the cells were able to again increase their level of intracellular Ca^2+^. The co-application of AC inhibitors, such as MDL-12,330A and SQ-22536, significantly reduced the Ca^2+^ response (Figure 3b; Supplementary Figure 1). A similar effect was observed with the protein kinase A (PKA) inhibitor H-89 (10 *μ*M) (Figure 3c). To exclude the involvement of a PLC-mediated pathway, we pre-incubated K562 cells for 30 min with 30 *μ*M of the PLC inhibitor U73122. U73122 had no significant effect on the isononyl alcohol-induced Ca^2+^ increase (Figure 3d). Next, we wanted to determine which downstream ion channels in the OR-mediated signaling pathway were involved in the calcium influx. In a previous study, the expression levels of several different L-type and T-type calcium channels in K562 cells were shown.^11^ In accordance with this, Manteniotis *et al*.^11^ found L-type calcium channels to be involved in the OR2AT4-mediated signaling pathway and to be most likely activated by PKA. In the present work, the same results could be generated for OR51B5. Blocking of either T-type or L-type calcium channels with NNC-55396 (10 *μ*M), L-cis diltiazem (150 *μ*M) a CNG channel blocker, that is also known to inhibit L-type calcium channels and Verapamil (20 *μ*M) significantly decreased the isononyl alcohol-induced Ca^2+^ response. In Figure 3f we summarized all of the measured data collected with the calcium imaging experiments in K562 cells.

### Isononyl alcohol activates an AC-mediated signaling pathway in native white blood cells of AML patients

**Figure 4:**
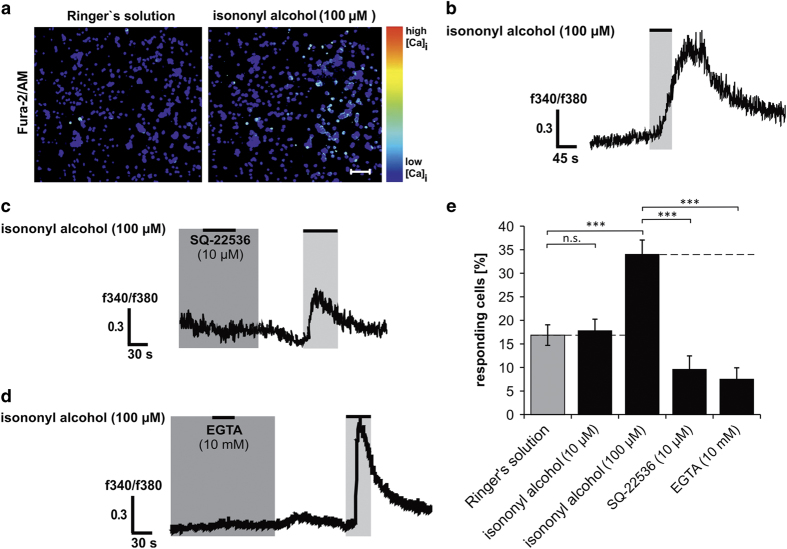
Calcium imaging experiments in the native blood samples of clinically diagnosed AML patients. (**a**) Native white blood cells from AML patients showed an increase in their intracellular Ca^2+^ levels during isononyl alcohol application. (**b**) Representative calcium imaging with the application of 100 *μ*M isononyl alcohol. (**c**) SQ-22536 abolished the isononyl alcohol-induced increase in intracellular Ca^2+^. (**d**) In absence of extracellular Ca^2+^ the isononyl alcohol-induced increase in intracellular Ca^2+^ was abolished. (**e**) Isononyl alcohol (100 *μ*M) led to a significant increase in intracellular Ca^2+^ in ~35% of all AML cells. SQ-22536 and the extracellular Ca^2+^ chelator EGTA reduced the number of responding cells.

To investigate whether this signaling pathway is also activated in native, undifferentiated myeloid cells, we isolated white blood cells from clinically diagnosed AML patients due to their relatively high levels of blast cells in the peripheral bloodstream. During the treatment with isononyl alcohol, the AML cells showed a rapid increase in their amount of intracellular Ca^2+^ with similar kinetics as those of K562 cells (Figures 4a and b). In total, 100 *μ*M isononyl alcohol could increase the cytosolic Ca^2+^ level of ~34% of all AML cells. To investigate whether the same signaling pathway as in K562 cells is involved, we used the same inhibitors that we used on K562 cells. Both the AC inhibitor SQ-22536 and depletion of extracellular Ca^2+^ significantly reduced the intracellular Ca^2+^ levels, thus validating the involvement of an AC-mediated signaling pathway downstream of the OR51B5 activation in AML patient blood cells (Figures 4c and d). An overview of the calcium imaging results obtained in AML cells is shown in Figure 4e.

### Isononyl alcohol alters the phosphorylation of MAPK in K562 cells

**Figure 5:**
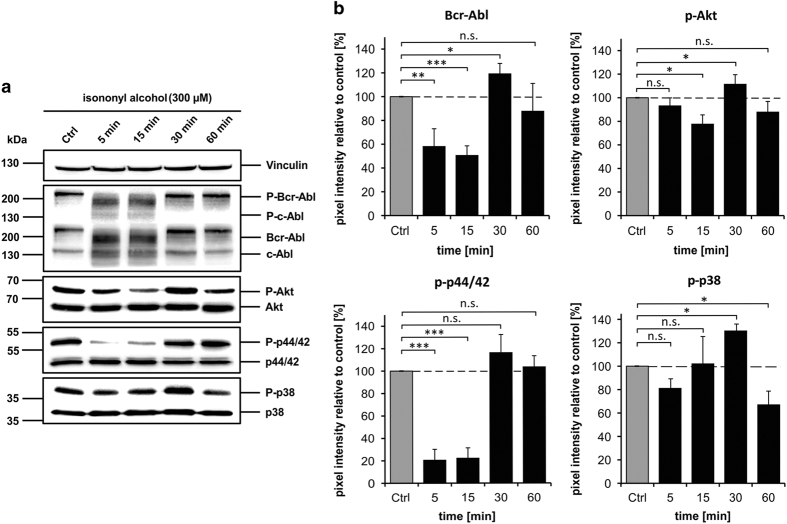
Examination of the protein kinase phosphorylation after isononyl alcohol application. (**a**) Exemplary western blots are shown for the alterations in the phosphorylation of protein kinases during isononyl alcohol incubation. Vinculin was used as a loading control. (**b**) Summarized results for the phosphorylation of various protein kinases. After 60 min of isononyl alcohol incubation, only p38-MAPK phosphorylation was significantly reduced.

An increase in intracellular Ca^2+^ is known to alter physiological properties, such as proliferation, apoptosis and differentiation, by altering the phosphorylation of MAPKs in a wide variety of cell types.^18–21^ However, the physiological involvement of MAPKs could vary depending on the cell system and the applied substance.^22,23^ In addition, it is widely known that CML is driven by an upregulation of the fusion protein *Bcr-Abl* in 95% of all patients.^24^ Therefore, using western blot experiments, we investigated the regulation of *Bcr-Abl* and MAPK phosphorylation after a 1 h incubation with 300 *μ*M isononyl alcohol (Figure 5a). In Figure 5b, we summarized the data regarding *Bcr-Abl,* Akt, p44/42 and p38-MAPK phosphorylation. Phosphorylation of *Bcr-Abl* is known to induce proliferation and apoptosis resistance.^24–26^ However, phosphorylation of *Bcr-Abl* was significantly downregulated after 5–15 min of incubation with isononyl alcohol (Figure 5b). After 30 min of incubation, *Bcr-Abl* phosphorylation returned to basal levels. A similar regulatory pattern was observed for p44/42-MAPK (Erk1/2), which is known to be involved in the apoptosis of K562 cells. JNK-MAPK phosphorylation was not affected by isononyl alcohol (data not shown). Akt phosphorylation, which is known to enhance cell survival, was significantly altered after 15–30 min, but not during later stages of isononyl alcohol incubation.

Interestingly, the phosphorylation of p38-MAPK was significantly reduced after 60 min of odor incubation. The downregulation of p38-MAPK phosphorylation is known to be involved in physiological effects such as proliferation.^27^

It is well known that intracellular Ca^2+^ can activate a variety of proteins. One such protein that activates many proteins after its phosphorylation is the calcium-calmodulin kinase 2 (CaMKII). Here, we showed that after CaMKII inhibition with the CaMKII inhibitor KN-62 the phosphorylation of p38-MAPK returned to basal levels (Supplementary Figure 2). This suggests that the activation of OR51B5, which leads to a Ca^2+^ influx, is responsible for the decreased p38-MAPK phosphorylation.

### Isononyl alcohol inhibits the proliferation of K562 cells

**Figure 6:**
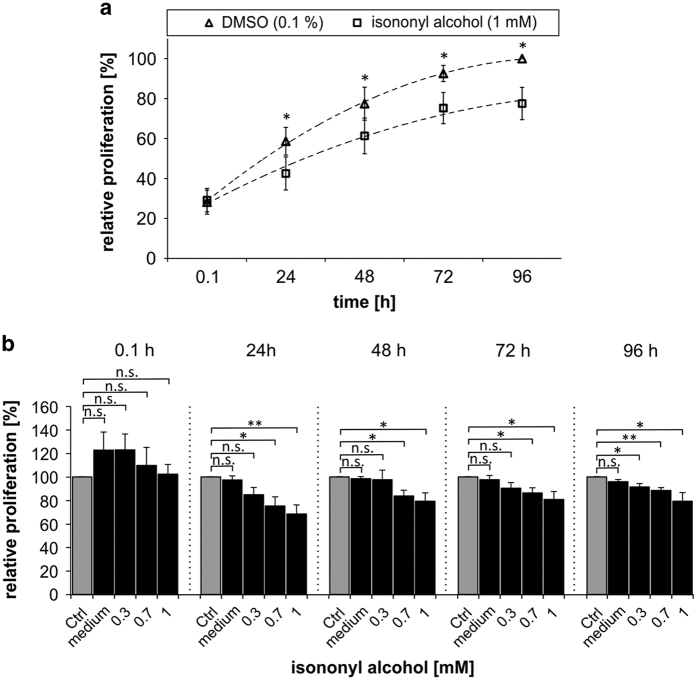
Isononyl alcohol decreases the proliferation of K562 cells. (**a**) Isononyl alcohol (1 mM) significantly decreased cell proliferation within 24 h. After 5 days the cell proliferation was reduced up to ~25% compared with the control cells. (**b**) Control cells (Ctrl: DMSO 0.1%) did not exhibit altered cell proliferation compared with cells cultured in DMSO-free RPMI medium. Isononyl alcohol is able to decrease cell proliferation in time- and concentration-dependent manners. The strongest effect was observed after 5 days of incubation with 1 mM isononyl alcohol.

To investigate whether the isononyl alcohol-induced alteration in the phosphorylation of p38-MAPK impacts cell proliferation, we used the CyQUANT Proliferation Assay and incubated K562 cells for 5 days with varying concentrations of isononyl alcohol (Figures 6a and b). K562 cell proliferation after treatment was compared with the control cells. The proliferation of K562 cells exposed to DMSO is not altered compared with the cells treated with medium only.^11^

However, within the first 24 h, 0.7–1 mM isononyl alcohol significantly reduced proliferation by ~20% (Figure 6a). After 5 days the K562 cell proliferation remained reduced by 25%. In addition, 300 *μ*M isononyl alcohol showed a significant effect after 5 days of incubation on the proliferation of K562 cells (Figure 6b).

## Discussion

In recent years, it has been shown several times that when ectopically expressed in different parts of the human body, olfactory receptors can alter physiological effects after their activation.^2,3,6–10,12^ ORs are expressed at high levels in cancer cells in the prostate, liver or intestine.^3,6,7^ ORs expressed in cancer cells can influence cellular functions, such as proliferation, apoptosis, migration or differentiation. For instance, in the prostate cancer cell line LNCaP and in primary prostate cancer cells, *β*-Ionone-induced OR activation caused an ~40% inhibition of cell proliferation.^6^ Similarly, another study reported a reduction in hepatocarcinoma cell proliferation mediated by the (−)-citronellal-induced activation of OR1A2.^7^ According to our investigations in hepatocarcinoma cells, the OR51B5-mediated increase in intracellular Ca^2+^ and MAPK phosphorylation is responsible for changes in various physiological processes.

Here, we report the ectopic expression of OR51B5 in the CML cell line K562 and in white blood cells derived from AML patients. We showed that the activation of OR51B5 leads to an intracellular Ca^2+^ increase in K562 cells and in white blood cells of AML patients. An increase in intracellular Ca^2+^ leads to alternations in cellular functions either by an indirect mechanism or via inhibition of MAPK phosphorylation.^7,10,11,28–31^ The characterization of the OR51B5-mediated signaling pathway showed that the receptor activates an AC-mediated pathway in both AML and CML and further activates downstream L-type and T-type calcium channels. Similarly, additional ectopically expressed ORs, such as OR2AT4 in CML, AML and in skin, or the OR1A2 in the hepatocarcinoma cell line Huh7, were reported to involve the AC-mediated signaling pathway.^7,10,11^ However, it is reported that ORs can use different mechanisms, such as activating Src kinases or using a Gq-, Gi-mediated pathway.^3,32–34^

Different concentrations of isononyl alcohol inhibit the proliferation of K562 cells by inhibiting the phosphorylation of p38-MAPK. Similarly, other studies in K562 cells showed that although an increase in p38-MAPK phosphorylation did not affect the cell proliferation, a decrease in p38-MAPK phosphorylation significantly reduced cell proliferation.^35^

In a previous study we observed the expression of several ORs in acute and chronic myelogenous leukemia.^11^ We characterized the activation of OR2AT4 by Sandalore in K562 cells and in AML blood samples of clinically diagnosed patients. In CML and AML, OR2AT4 is involved in the inhibition of proliferation, the enhancement of cell apoptosis and the differentiation to hemoglobin carrying cells. In the current work, OR51B5 significantly increases the levels of intracellular Ca^2+^ through an AC-cAMP-mediated pathway and downstream L-type and T-type calcium channels. This signaling pathway is similar to the pathway that has been shown to downstream of OR2AT4 in K562 cells. However, the EC_50_ of isononyl alcohol for OR51B5 is higher than the EC_50_ shown for OR2AT4. However, it is comparable to the reported EC_50_ of (−)-citronellal for OR1A2 in Huh7 cells.^7^ This suggests that isononyl alcohol is a low-affinity agonist, which could explain the lower Ca^2+^ influx and different physiological effects induced by OR51B5. It seems that OR ligands with higher affinities would exert a more significant impact on physiological processes.

For example, the activation of OR51B5 by 0.3–1 mM isononyl alcohol caused an inhibition of K562 cell proliferation of ~25%. In contrast, the activation of OR2AT4 in K562 cells led to a complete inhibition of cancer cells proliferation. The reason for this discrepancy may be the varying levels of Ca^2+^ influx, which in turn activates various downstream signaling pathways by phosphorylating MAPKs, Akt or *Bcr-Abl*.

However, in accordance with our previous study, a decreased p38-MAPK phosphorylation could be the reason for the OR51B5-mediated inhibition of proliferation.

Our data suggest that olfactory receptors expressed in CML and AML are involved in major physiological mechanisms and thus can be considered as new and promising pharmacological anti-leukemic drug targets for alternative treatments. Several studies describe the anti-cancer effects induced by odorous substances, such as terpenes, which are components of essential oils.^7,36–41^ In total, half of all produced anti-cancer drugs are composed of natural products or synthetic derivatives.^42,43^ Currently, supportive care and allogeneic transplantation of AML is cured in <5% of patients under 60 years of age.^44,45^ Over the past 30 years, the therapeutic treatment of AML has not significantly improved.^45^ Little is known regarding potential pharmacological approaches possibilities to treat this disease, indicating an urgent need for novel treatment modalities.

## Materials and Methods

### Cell culture

K562 cells were purchased from LGC Standards GmbH (Wesel, Germany). The cells were cultured at 37 °C and 6% CO_2_ in RPMI-1640 medium (Life Technologies, Carlsbad, CA, USA) containing 10% (v/v) fetal bovine serum (FBS), 5% GlutaMAX (Life Technologies) and 100 units/ml penicillin/streptomycin.^17^ The cells were diluted 1:10 three times per week using freshly prepared medium. Depending on the experiments, the cells were counted and seeded at densities of 1×10^5^ cells/ml to 1×10^6^ cells/ml. All of the odorants (Sigma-Aldrich, Munich, Germany) were dissolved in DMSO to create stock solutions, which were diluted in medium to a maximal DMSO concentration of 0.1%. Odor-free medium containing 0.1% DMSO was used in all our experiments as a negative control. To account for any influence of 0.1% DMSO in the medium, we also performed additional control experiments with DMSO-free medium.

### Patient samples

The analysis of blood samples from AML patients was approved by the ethics committees of the University Hospital Essen and of the Knappschaftskrankenhaus Bochum (ZOKS 3841-10).The patients gave informed consent according to institutional guidelines. In total, 25 patients with various types of AML participated in the study. Blood was drawn from central venous lines. For measuring the effects in AML blood samples, each patient sample was handled separately. The blood samples were incubated with Lymphoprep (Stemcell Technologies, Colonge, Germany) according to the manufacturer’s instructions. Erythrocytes were eliminated with 5 min of incubation with Lysing Buffer (BD Biosciences, Heidelberg, Germany) and subsequent centrifugation.

### Cell proliferation

The cells were seeded into cell culture flasks at a density of 1×10^5^ cells/ml and treated with varying odorant concentrations (10–300 *μ*M) or 0.1% DMSO as a control. Alternatively, the cells were stimultaneously stimulated with the odor and inhibitors against p38-MAPK, Akt and p44/42-MAPK. To measure the cell viability, a proliferation assay was performed every 24 h in 96-well plates using the CyQUANT cell proliferation kit (Life Technologies) according to the manufacturer’s protocol. The cell proliferation was measured for 5 days.

### RNA isolation and PCR

Total RNA was extracted from K562 cells using the RNEasy Mini Kit (Qiagen, Hilden, Germany) according to the manufacturer’s instructions. Subsequently, DNase I treatment was performed with the TURBO DNA-free Kit (Life Technologies) before complementary DNA (cDNA) was synthesized using the iScript cDNA Synthesis Kit (Bio-Rad Laboratories, Hercules, CA, USA). PCR was conducted with cDNA (the equivalent of 250 ng of total RNA) and specific primer pairs of the following sequences:

OR51B4 forward: 5′CAATGGCACCCTCCTTCTTC3′

OR51B5 reverse: 5′CAAGCAGAATGCCAGACTCG3′

TBP forward: 5′TATAATCCCAAGCGGTTTGC3′

TBP reverse: 5′GCTGGAAAACCCAACTTCTG3′

GAPDH forward: 5′ACCACAGTCCATGCCATCAC3′

GAPDH reverse: 5′TCCCACCACCCTGTTGCTGTA3′

RT-PCR and quantitative real-time PCR (qPCR) were performed with the GoTaq qPCR Master Mix (Promega, Madison, WI, USA). The PCR reactions were performed using the Mastercycler ep realplex (Eppendorf, Hamburg, Germany) with the following cycle profile:

5 min at 95 °C followed by 40 cycles of 45 s at 95 °C, 45 s at 60 °C (for OR51B5, TBP and GAPDH), 45 s at 72 °C and a final extension of 10 min at 72 °C.

The evaluation of the qPCR was performed using the ΔΔCT method as previously.^11^

### Flow cytometry

Erythroid differentiation was assayed by staining K562 cells with an anti-CD235a antibody (Monoclonal Mouse Anti-Human CD235a Glycoporin A/FITC and A/RPE, Clone JC159) after 6–10 days of odor incubation. Flow cytometric data acquisition was performed with an LSRII flow cytometer (BD Bioscience), and the data were analyzed by DIVA (BD Bioscience) and FlowJo software (Treestar, Ashland, OR, USA).

### Calcium imaging

K562 cells were incubated for 20 min on poly-l-Lysine (Sigma-Aldrich) coated 35-mm dishes with RPMI-1640 medium (Life Technologies), with 10% FBS, 5% GlutaMAX and 100 units/ml penicillin/streptomycin and incubated for a further 20 min in RPMI-1640 containing 3 *μ*M Fura-2-AM (Molecular Probes, Eugene, Oregon) at 37 °C and 6% CO_2_. White blood cells derived from AML patients were incubated for 20 min on concanavalin A (Sigma-Aldrich) laminated 35-mm dishes. Before assaying the cells, the RPMI-1640 medium was replaced with Ringer’s solution (140 mM NaCl, 5 mM KCl, 2 mM CaCl_2_, 1 mM MgCl_2_ and 10 mM HEPES; pH 7.3). The calcium imaging experiments were performed as described previously.^2,6^ To create Ca^2+^-free conditions, we added 10 mM EGTA into a Ca^2+^-free Ringer’s solution. We used the adenylate cyclase inhibitors MDL-12,330 A (10 *μ*M), and SQ-22536 (10 *μ*M), the phospholipase C inhibitor U73122 (30 *μ*M), and the protein kinase A inhibitor H-89 (10 *μ*M) to analyze the OR51B5-mediated signaling pathway (all inhibitors were purchased from Sigma-Aldrich). Furthermore, we used various calcium channel blockers, such as NNC-550396 (30 *μ*M) that inhibits T-type calcium channels, and L-cis diltiazem (150 *μ*M) that blocks CNGA1 and CNGA3 channels as well as L-type calcium channels (Sigma-Aldrich). Before applying the odorant, the cells were pre- with the blocking solution. To investigate the cell viability 100 *μ*M ADP was applied to the cells at the end of each experiment. In previous studies, ADP has been shown to increase the amount of intracellular Ca^2+^ in K562 cells.^46,47^

### Western blotting analysis

K562 cells were treated with 300 *μ*M isononyl alcohol for varying amount of time (5, 15, 30 or 60 min) at 37 °C and 6% CO_2_. The cells were harvested and homogenized in lysis buffer (RIPA: 50 mM Tris-HCl, 150 mM NaCl, 1 mM EDTA, 1% Triton X-100, pH 7.4) with protease inhibitor (cOmplete, Roche Applied Science, Basel, Switzerland) and phosphatase inhibitor (PhosSTOP, Roche Applied Science). All western blot experiments were performed as previously described.^6,10^ The detection was performed using the ECL Select Western Blotting Detection System (Amersham Biosciences, GE, Healthcare, Solingen, Germany). We used the polyclonal rabbit antibodies against p44/42-MAPK (cat. no. #9102), p38-MAPK (cat. no. #9212), JNK-SAPK (cat. no. #9252), Akt (cat. no. #4059) and Bcr-Abl (cat. no. #2862), as well as the phosphor-specific antibodies against pp44/42-MAPK (cat. no. #9101), pp38-MAPK (cat. no. #9211), pJNK-SAPK (cat. no. #9251), Bcr-Abl (cat. no. #2864) and pAkt (cat. no. #4060) (Cell Signaling Technology). For quantification, we used the Java-based software ImageJ 1.46 to calculate the relative pixel intensity.^48^ To investigate the effects of the intracellular Ca^2+^ increase, we inhibited calmodulin kinase II (CaMKII) with KN-62 (10 *μ*M) (Sigma-Aldrich). KN-62 binds directly to CaMKII and leads to its inactivation.^49^ Because CaMKII is activated by Ca^2+^ and is necessary for the activation of calmodulin, use of this inhibitor allows for investigation of the effects of increased intracellular Ca^2+^. Therefore, we pre-incubated the cells with KN-62 for at least 60 min prior to incubation with isononyl alcohol. The effects of KN-62 on isononyl alcohol-treated cells were observed for a further 60 min after which time the proteins were isolated. To load equal amounts of protein, we determined the total protein amount using the Bradford assay (Life Technologies) and optimized the protein loading with Coomassie-staining of SDS-PAGE gels. We used an antibody against the housekeeping protein vinculin (Cell Signaling Technology) as a control in some experiments.

### Immunocytochemistry

For immunocytochemistry experiments, the following antibodies were used: a custom-designed antibody against the C terminus of OR51B5, polyclonal, dilution 1:50 (Eurogentec, Seraing, Belgium); an anti-OR51B5 antibody, polyclonal, dilution 1:25 (Eurogentec); and an anti-caspase-3 antibody, polyclonal, dilution 1:300 (Sigma-Aldrich). Briefly, 5-mm cover slips were coated for 15 min with poly-L-lysine (Sigm-Aldrich) and air-dried for 30 min. Next, 1–5×10^6^ per ml K562 cells in 60 *μ*l were incubated in medium on the coated 5-mm cover slips. The medium was removed, and the cells were fixed in 4% paraformaldehyde for 20 min at 4 °C. To wash and permeabilize the membrane, PBS medium including 0.1% Triton X-100 (Sigma-Aldrich) (PBST) was used. The K562 cells were incubated with primary antibodies, diluted in blocking solution and containing PBST, 1% fish gelatin and 3% horse serum for 3 h at room temperature. After having rewashed the cells three times with PBST, the cells were incubated with a secondary goat anti-rabbit antibody (1:1000) (Alexa Fluor 488, Life Technologies) and 4ʹ6ʹ-diamidino-2-phenylindole (1:300) in blocking solution for 1 h at room temperature. Next, the cells were washed again with PBST and mounted on a slide with ProLong Gold Antifade Reagent (Invitrogen). Images were obtained using a Zeiss LSM 510 META confocal microscope (Carl Zeiss, Germany).

### Statistical analysis

Statistical analyses were performed with Microsoft Excel and Sigma Plot 12. All results were tested for normality (Shapiro–Wilk test) and equal variance. The significance levels were calculated with a two-tailed unpaired *t*-test, and significant values classified as <0.05 (*),<0.01 (**) and<0.0005 (***). Dose–response curves and EC_50_ values were calculated using the 4-parameter Hill model. The data were represented as the mean±S.E.M. (standard error of the mean) from at least three independent experiments.
