# Supplementary material for: Deorphanization and characterization of the ectopically expressed olfactory receptor OR51B5 in myelogenous leukemia cells
Source: Cell Death Discov. 2016 May 9;2:16010–. doi: 10.1038/cddiscovery.2016.10 (PMC4979495; doi:10.1038/cddiscovery.2016.10)
Supplement: Supplementary Information [file cddiscovery201610-s1.doc]

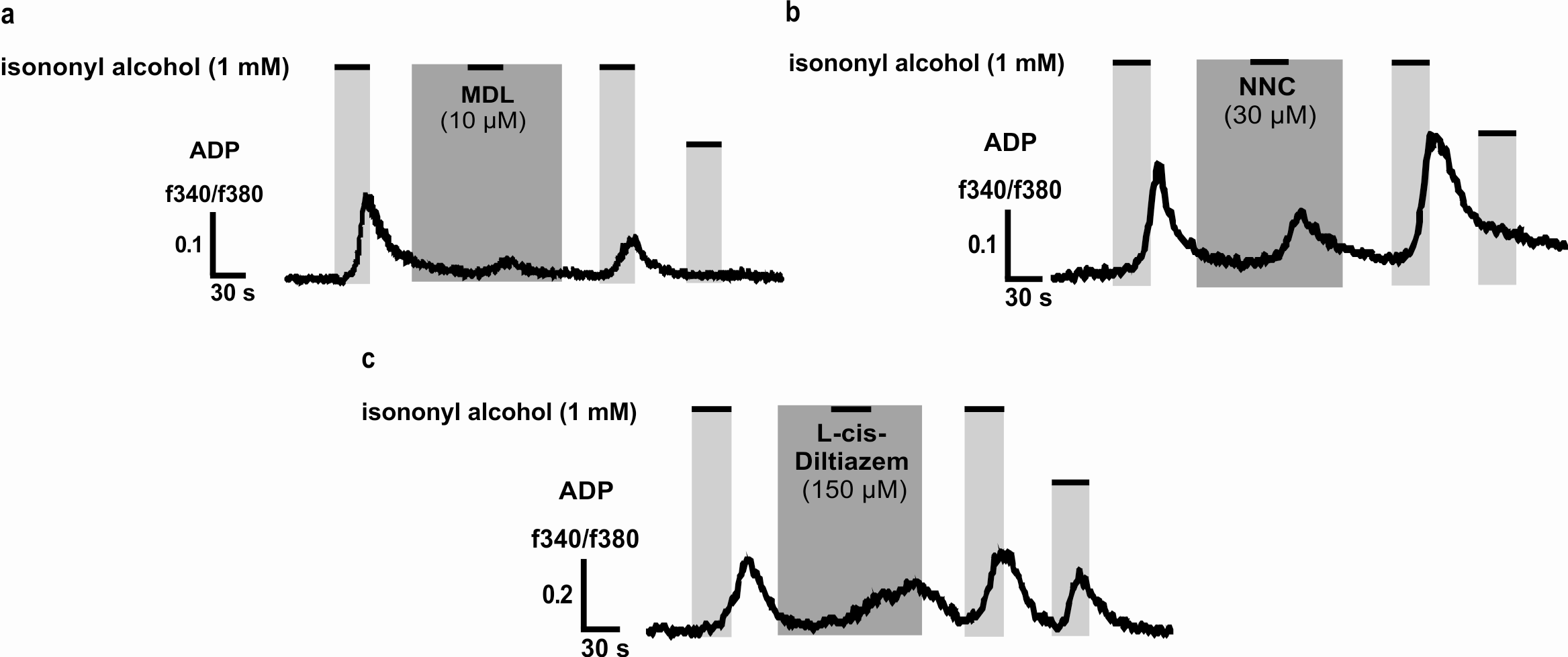


**Supplementary Figure 1.** **(a)** As shown for SQ-22536, also 10 µM MDL abolished the isononyl alcohol induced increase of intracellular calcium and approved the involvement of the AC in the OR51B5 mediated signaling transduction. **(b)** NNC-550396 (30 µM), a T-type calcium channel blocker, significantly and reversibly reduced the isononyl alcohol induced increasement of intracellular calcium. **(c)** L-cis diltiazem (150 µM) that is known to inhibit CNGA1 and CNGA3 as well as L-type calcium channels significantly reduced the calcium respond after isononyl alcohol application in K562.


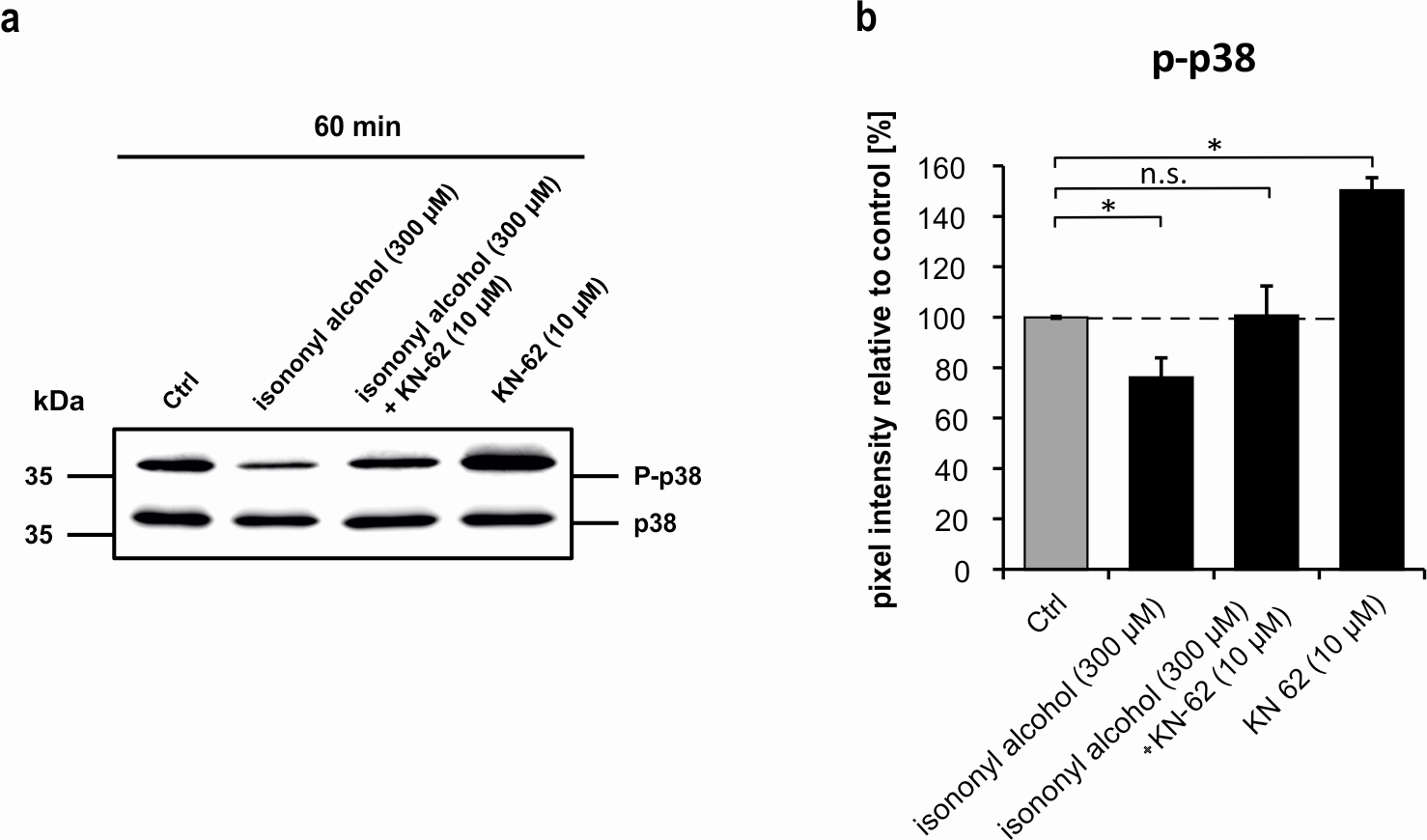


**Supplementary Figure 2. (a)** CaMKII inhibition with the CaMKII inhibitor KN-62 returned the phosphorylation of p38-MAPK to basal levels. This suggests that the activation of OR51B5, which leads to a Ca^2+^ influx, is responsible for the decreased p38-MAPK phosphorylation. **(b)** Summarized western blots for the involvement of CaMKII in the regulation of the p38-MAPK phosphorylation induced by isononyl alcohol.
